# Impact of pulse modulation technology on operating room time and blood loss in HoLEP: real-world data from an academic center

**DOI:** 10.1007/s10103-026-04969-3

**Published:** 2026-07-29

**Authors:** Juanita Velasquez-Ospina, Sabika Sadiq, Ansh Bhatia, Haşim Bakbak, Ahmad Abdelaziz, Gurpremjit Singh, Archan Khandekar, Jonathan Katz, Robert Marcovich, Hemendra N. Shah

**Affiliations:** 1 Desai Sethi Urology Institute, University of Miami Miller School of Medicine, Miami, Florida USA; 2 Department of Radiology, University of Miami, Coral Gables, Florida USA

**Keywords:** BPH, Laser, Holmium, HoLEP, MOSES

## Abstract

Purpose. To compare perioperative and functional outcomes of standard holmium, Moses 1.0, and Moses 2.0 laser enucleation performed at an academic center with trainee involvement. Methods. We retrospectively reviewed men undergoing HoLEP between June 2022 and May 2024. Patients were grouped by laser platform: standard holmium, Moses 1.0, or Moses 2.0. Baseline demographics, perioperative data, complications, and functional outcomes up to six months were analyzed using analysis of variance or Kruskal–Wallis tests. Results. Among 389 patients (standard holmium 206; Moses 1.0 126; Moses 2.0 57), baseline characteristics were similar. Median operative times were 95, 105, and 100 min for standard holmium, Moses 1.0, and Moses 2.0, respectively (p = 0.25), and median prostate weights were comparable (84 g, 85 g, 79 g; p = 0.44). Hospital stay was slightly longer with Moses lasers (1.6 vs. 1.4 days, p < 0.001), though this difference is unlikely to be clinically significant. Objective measures of bleeding—postoperative hemoglobin drop (1.7–2.0 g/dL) and transfusion rates (0–1.6%)—did not differ significantly between groups. Postoperative catheter duration, hospital stay, and complication rates were also comparable. Functional outcomes improved in all groups through 6 months, with no intergroup differences. Conclusion. HoLEP is safe and effective across conventional holmium, Moses 1.0, and Moses 2.0 platforms, with comparable perioperative and functional outcomes. Although the literature suggests that Moses 2.0 may facilitate same-day discharge, its absence should not limit HoLEP availability, and platform selection may reasonably reflect institutional resources and surgeon preference.

## Introduction

Benign prostatic hyperplasia (BPH) is common in aging men, increasing with rising life expectancies. According to the Global Burden of Disease 2021 study, approximately 112.5 million men worldwide were living with BPH, accounting for an estimated 2.24 million disability-adjusted life-years (DALYs) [1]. Management ranges from lifestyle modification and medication to surgery, typically reserved for refractory cases [2]. While transurethral resection of the prostate (TURP) remains the historic gold standard, newer technologies continue to evolve with distinct advantages and trade-offs [3, 4].

Holmium laser enucleation of the prostate (HoLEP) has emerged as a highly effective, minimally invasive surgical option [3]. Standard holmium: YAG lasers deliver a single energy pulse with significant attenuation as it traverses the aqueous medium between fiber and tissue [5]. Moses 1.0 pulse modulation divides the pulse into two sequential sub-pulses: the first creates a vapor channel, and the second delivers energy through it with reduced attenuation [6, 7]. Originally developed to optimize lithotripsy, Moses 1.0 featured two manually selected modes (Contact and Distance) based on fiber-to-target distance [5, 6]. Moses 2.0 further refined the dual-pulse technology for soft tissue applications and expanded the available frequency range up to 120 Hz, enabling greater efficiency and procedural speed. Several studies have demonstrated improved enucleation, operative and hemostasis times with Moses technology, with similar safety profiles to standard holmium [7–9]. However, functional outcomes such as symptom improvement remain comparable across platforms [10, 11]. Moreover, most studies have been limited to two-arms (holmium vs. Moses 1.0 or Moses 2.0) and largely performed in expert-only settings [7–12]. 

Limited data exist comparing all three laser modalities in a single cohort, particularly in real-world academic environments. This study addresses these gaps by evaluating the efficacy and safety of en-bloc HoLEP using standard holmium, Moses 1.0, and Moses 2.0 lasers, including cases with trainee participation. To the best of our knowledge, this is the first study comparing these three laser platforms in a single cohort.

## Materials and methods

### Study design and population

Following IRB approval (20180511), we conducted a retrospective review of men undergoing HoLEP at our institution between June 2022 and May 2024. Patients were grouped by laser platform based on operating room (OR) assignment: standard holmium, Moses 1.0, or Moses 2.0 (Lumenis Pulse™ 120 H Holmium Laser System with MOSES™ Technology). We excluded patients with neurogenic bladder, urethral stricture, bladder cancer, prior radiation for localized prostate cancer (PCa), or channel procedure for bladder outlet obstruction for advanced PCa. Those with low-risk PCa on active surveillance undergoing HoLEP for bladder outlet obstruction were included.

### Intervention

Enucleation was performed using a 26-Fr laser endoscope (Karl Storz, Tuttlingen, Germany) with a 550-µm fiber at 2 J/30 Hz across all platforms, consistent with literature showing no difference in safety or efficacy between low- and high-power HoLEP [13, 14]. Morcellation was performed with either the VersaCut™ Tissue Morcellator System or the Piranha Morcellator (Richard Wolf, Vernon Hills, IL, USA). All procedures followed the en-bloc technique by an experienced endourologist, with graded trainee involvement following a structured, competency-based progression system.

### Postoperative care and follow-up

A 22-Fr Foley catheter was placed without traction, with continuous bladder irrigation maintained overnight. Hemoglobin was obtained on postoperative day 1 (POD1). Discharge was planned on POD1 following a successful voiding trial. Patients were followed at 6 weeks, 3 months, and 6 months. Outcomes assessed included International Prostate Symptom Score (IPSS), maximum urinary flow rate (Qmax), post-void residual (PVR), and continence (defined as any postoperative involuntary urine leakage requiring protection at any time). Prostate-specific antigen (PSA) nadir was measured at 3 months.

### Data collected

Collected variables included age, body mass index (BMI), comorbidities (hypertension and diabetes mellitus), prostate size (determined by ultrasound, CT, or MRI), PSA, IPSS, Qmax, and PVR. Patients with elevated PSA underwent MRI, with further management determined by shared decision-making. Perioperative outcomes included hemoglobin drop, total operative time (total time spent by the patient in the OR), resected prostate weight, catheter duration, and hospital stay. Complications assessed included blood transfusion, acute urinary retention (AUR) on POD1 following Foley catheter removal, urinary incontinence, and urethral strictures. Functional outcomes were defined as changes in IPSS, Qmax, and PVR.

### Statistical analysis

Continuous variables were reported as mean ± SD or median [IQR], and categorical as n (%). Group comparisons were performed using three-way ANOVA or Kruskal–Wallis tests, with *p* < 0.05 considered statistically significant. Analyses were conducted in RStudio (v4.1.3). Missing data were analyzed on available-case basis.

## Results

### Patient characteristics

A total of 389 patients underwent HoLEP: 126 with Moses 1.0, 57 with Moses 2.0, and 206 with standard holmium. Baseline demographics were mostly comparable. (Table 1)

**Table 1:** Patient characteristics, perioperative outcomes, and follow-up functional data

	MOSES 1		MOSES 2		Standard HoLEP		*p*-value
Baseline demographics and preoperative characteristics							
N	126		57		206		
Age	71.6 ± 7.4		71.5 ± 7.6		74.2 ± 8.2		0.84
Prostate size	135.5 ± 74.3		122.2 ± 72.4		124.2 ± 68.3		0.96
BMI	28.1 ± 4.2		27.6 ± 4.5		27.7 ± 4.3		0.35
DM	29.4%		31.6%		18.4%		**0.02**
HTN	89%		87.7%		85.4%		0.74
PSA	7.0 ± 6.2		6.6 ± 5.2		6.8 ± 8.3		0.09
Intraoperative and immediate postoperative characteristics							
Median operating room time (mins)	105 [80–157]		100 [37–163]		95 [73–115]		0.25
Median hospital stay (days)	1.6 ± 0.8		1.6 ± 0.4		1.4 ± 2.1		**< 0.001**
Resected prostate volume (grams)	85.1 [58–123]		79 [44–113]		84 [66–116]		0.44
Hb drop (g/dL)	2 [1.1–2.9]		1.7 [1.1–2.4]		2 [1.2–2.9]		0.13
Perioperative adverse events and early outcomes							
AUR on POD1	4.8%		10.5%		7.8%		0.34
Blood transfusion	1.6%		0%		0.5%		0.73
Incontinence at last follow-up	53.2%		43.9%		56.8%		0.217
Urethral stricture and/or BNS	4%		3.6%		3.4%		0.93
Intermediate follow-up functional outcomes*							
IPSS							
Baseline	*n* = 94	18.5 [13–23]	*n* = 48	16 [11–21]	*n* = 168	18 [13–22]	0.14
3 months	*n* = 67	4 [1–7]	*n* = 8	3 [1–6]	*n* = 105	3 [1–7]	0.06
6 months	*n* = 19	3 [1–4]	*n* = 2	4.5 [4–5]	*n* = 67	2 [1–3]	0.15
Qmax							
Baseline	*n* = 86	8.1 [6–9]	*n* = 39	7.5 [6–8]	*n* = 146	7.5 [5–10]	0.16
3 months	*n* = 59	21 [11–28]	*n* = 25	21 [16–26]	*n* = 142	17 [11–26]	0.16
6 months	*n* = 21	14 [7–25]	*n* = 5	23 [11–25]	*n* = 79	16 [14–19]	0.29
PVR							
Baseline	*n* = 97	128 [46–254]	*n* = 49	140 [38–243]	*n* = 163	141 [67–260]	0.70
3 months	*n* = 61	19 [0–50]	*n* = 25	16 [1–44]	*n* = 146	17 [0–40]	0.14
6 months	*n* = 21	4 [0–28]	*n* = 7	40 [3.5–49]	*n* = 86	13 [0–50]	0.45

*BMI* body mass index, *DM* diabetes mellitus, *HTN* hypertension, *PSA* prostate-specific antigen, *Hb* hemoglobin, *IPSS* International Prostate Symptom Score, *PVR* Post-void residual volume, *Qmax* Maximum urinary flow, *AUR* acute urinary retention, *POD1* postoperative day 1, *BNS* bladder neck stenosis

*The *n* from the preceding column for each group represents the number of patients with available follow-up data

### Intraoperative and immediate postoperative outcomes

Median OR time and resected prostate volume were similar. Hemoglobin drop did not differ significantly: 2 g/dL Moses 1.0 and standard holmium; 1.7 g/dL Moses 2.0. Hospital stay differed statistically but not clinically.

### Complications

AUR on POD1 and transfusion rates were similar across groups. Urinary incontinence and urethral stricture/bladder neck stenosis rates showed no differences.

### Functional outcomes

Baseline functional measures were comparable. IPSS improved in all groups through 6 months, without intergroup differences. Qmax increased from 7 to 8 mL/s to 17–23 mL/s at 3 months and remained improved at 6 months, except for a mild decline in Moses 1.0 (*p* = 0.29). PVR decreased from 128–141mL to < 20mL at 3 months, with further reductions by 6 months in Moses 1.0 and standard holmium, while increasing slightly to 40mL in Moses 2.0.

## Discussion

### Study overview

The concept of pulse modulation for HoLEP is appealing to urologists, and prior studies have suggested efficiency gains with Moses technology (Table 2). We compared perioperative and functional outcomes using standard holmium, Moses 1.0, and Moses 2.0 in an academic setting with trainee involvement. Baseline characteristics were largely comparable across groups, although diabetes mellitus was more prevalent in the Moses groups. The high prevalence of comorbidities such as hypertension likely reflects the tertiary referral nature of our center, where patients with more complex medical histories are frequently treated. We found operative time and objective bleeding outcomes—including hemoglobin drop and transfusion rates—were similar across platforms.

**Table 2:** Review of literature on publications on pulse modulation in anatomic enucleation of the prostate

Author (year); Country; Study design	Group (*n*)	Preop prostate size	Enucleation time (min)	Hemostasis time (min)	Morcellation time (min)	Enucleation efficiency (g/min)	Enucleated weight (g)	Catheter duration	LOS	Hb drop ^a^ / HCT change
Large et al. (2019); USA; Retrospective review of prospective database Single surgeon, 2–3 lobe bottom-up, apical energy reduced.	Holmium Slimline 550 μm (50)	110.5 ± 85.5	22.5 ± 7.3 *	10.6 ± 6.1 *	11.6 ± 10.9 *	-	72.5 (3-265)	-	SDD: 0% *	1.5 + 1.2 *
	Holmium Slimline 1000 μm (50)	118.3 ± 92.4	16.4 ± 6.9 *	7.7 ± 5.2 *	10.2 ± 13.5 *	-	65.9 (4-315)	-	SDD: 8% *	1.5 + 1.6 *
	MOSES 1.0 550 μm (50)	155.6 ± 50.3	18.1 ± 8.6 *	6.3 ± 4.8 *	10.3 ± 13.4 *	-	76.7 (4-448)	-	SDD: 6% *	1 + 1.1 *
Mohammed et al. (2020); Italy; Prospective randomized trial Single surgeon, 3-lobe technique.	HoLEP (70)	84	30.5 *	5.9	11.7	-	-	-	2.8 days	1.01
	Moses 1.0 MoLEP (70)	85	27.1 *	5.3	13.2	-	-	-	2.7 days	0.95
Nevo et al. (2021); USA; Double blind RCT 2 surgeons + 2 trainees, lobe randomization.	Non-Moses HoLEP (27)	107 ^b^	50.1	6.5 *	-	0.7	-	-	-	-
	Moses 2.0 HoLEP (27)	107 ^b^	45.4	3.5 *	-	1.1	-	-	-	-
Nottingham et al. (2021); USA; Retrospective 2 endourologists, bottom-up technique.	Standard Holmium (50)	-	47.1	10.6 * ^c^	11.6	0.73	73	-	POD0 discharge 0%; POD1 46 (92%); POD2 4 (8%)	-
	Moses 2.0 MoLEP (54)	-	45.9	8.1 * ^c^	10.4	0.57	77	-	POD0 discharge 37 (68.5%); POD1 17 (31.5%); POD2 0%	-
Kavoussi et al. (2021); USA; Double blind RCT Single surgeon, 2-lobe bottom-up technique.	Standard HoLEP (30)	153 ± 58	80 ± 19 *	29 ± 15 *	16 ± 10	-	76 ± 28	1.1 ± 0.5 days	1.2 ± 0.61	HCT: -9 ± 4.6 *
	Moses 1.0 MoLEP (30)	131 ± 41	68 ± 20 *	19 ± 8 *	14 ± 10	-	65 ± 29	1.2 ± 0.63 days	1.1 ± 0.64	HCT: -6.4 ± 4.1 *
Noureldin et al. (2022); Canada; Retrospective review of prospectively collected data Single surgeon, top-down technique.	HoLEP (28)	115.6 ± 38.5	63.4 ± 17.8 *	7.1 ± 2.6 *	14.1 ± 7 *	1.3 ± 0.4 *	82.3 ± 41.2	-	-	14.7 ± 5 *
	MoLEP (62)	109.5 ± 30.8	47 ± 12.5*	3 ± 1.1 *	10.2 ± 5 *	1.7 ± 0.6*	78.5 ± 29.1	-	-	10.7 ± 4.5 *
Socarras et al. (2022); Spain; Single-arm prospective study using a historical control Single surgeon, En-bloc technique.	Non-Moses HoLEP (137)	75.77 ± 42.25	31.46 ± 14.85 *	8.35 ± 5.38 *	6.93 ± 6.64	2.54 ± 1.31 *	53 ± 35.94	17.35 ± 11.05 h	22.01 ± 5.71 h	1.73 ± 0.61
	Moses 2.0 MoLEP (80)	86.66 ± 50.0	22.1 ± 9.27 *	3.01 ± 2.5 *	6.16 ± 5.4	4.11 ± 2.41 *	56. 27 ± 36.93	17.9 ± 10.56 h	21.14 ± 6.41 h	1.53 ± 0.57
Tong et al. (2024); China; Single blind parallel RCT Single surgeon, standardized enucleation	Non-Moses HoLEP (42)	86.7 ± 18.5	27.2 ± 9.4	11.2 ± 5.1 *	-	2.6 ± 0.7	70.1 ± 18.3	1.4 ± 0.2 days	1.1 ± 0.1 days	-
	MoLEP (38)	92.2 ± 19.6	22.5 ± 7.6	6.6 ± 4.2 *	-	3.5 ± 0.8	73.6 ± 20.5	1.3 ± 0.1 days	1 ± 0.1 days	-
Hartung et al. (2024); Germany; Retrospective cohort study Single surgeon, En-bloc, bipolar cautery for hemostasis	HoLEP (117)	102 ± 53	29.3 ± 15.9	7.8 ± 4.6	17.3 ± 17.4	2.5 ± 1.3	73.3 ± 53.5	1.9 ± 0.8 days	3.7 ± 1.3 days	0.9 ± 1.1
	Moses 2.0 MoLEP (117)	104 ± 59	27.4 ± 16.3	8 ± 4.9	15.4 ± 11.2	2.6 ± 1.2	70.4 ± 51.7	1.9 ± 1 days	3.7 ± 1.7 days	0.7 ± 2.1
Elmansy et al. (2024); Canada; Retrospective review Single surgeon, top-down technique.	Moses 1.0 HoLEP (146)	109 (50–325)	52.5 (19–135) *	8 (4–16) *	11 (2–70)	1.63 (0.6–3.37) *	82 (18–303)	3 h (91.8% pts)	4 h: 91.1% pt 6 h: 0.7% pt < 24 h: 7.5% pt 48 h: 0.7% pt	11 g/L (range: 2–66)
	Moses 2.0 HoLEP (50)	117.5 (60–300)	42.5 (18–87) *	6 (2–11) *	12 (5–40)	2.2 (1.1–3.3) *	97.5 (32–264)	3 h (94% pts)	4 h: 94% pt 6 h: 0% pt < 24 h: 6% pt 48 h: 0% pt	9 g/L (range: 1–22)

Legend: *statistical significance difference demonstrated in the study

^a^ Hemoglobin reported as mg/dL unless otherwise specified

^b^ reported for the whole cohort, not specified between groups

^c^ specifically reported as “hemostasis pedal time in minutes”

*LOS* length of stay, *Hb* Hemoglobin, *HCT* hematocrit, *USA* United States of America, *HoLEP* Holmium laser enucleation of prostate, *MoLEP* holmium laser enucleation of the prostate using Moses technology, *RCT* Randomized controlled trial, *MoLEP* Moses laser enucleation of the prostate, *pts* patients, *POD* Post operative day, *SDD* same day discharge

### Operation time as a surrogate marker of efficiency

Since the primary goal of the Moses effect is to improve efficiency, shorter operative times would be expected compared with standard holmium. Nottingham et al. reported 91 vs. 81 min (standard holmium vs. Moses 2.0), and Kavoussi reported 126 vs. 101 min (Moses 1.0), with significance only in the latter [7, 15]. Neither study defined operative time explicitly. In Kavoussi et al.’s, the shorter time may reflect smaller prostate size in the Moses group (131 ± 41 vs. 158 ± 58 cc) [15]. This can be assumed as enucleation efficiency (weight of prostate tissue removed/enucleation time), which was not reported in the study, was similar in both study arms, calculated at 0.95 g/min.

OR time is a standardized metric recorded across institutions and reflects the entire surgical workflow, from anesthesia induction and patient positioning to enucleation, hemostasis, morcellation, and recovery. Although it does not isolate the laser-dependent phases of HoLEP, OR time captures the overall procedural duration and therefore represents a clinically relevant measure of OR utilization and perioperative efficiency.

In our cohort, total OR time—including surgical setup, anesthesia induction, operative time, and patient recovery—did not differ significantly among groups. While operative time is often used as a proxy for new surgical technology, it is an incomplete and misleading metric. Shorter operative times are not direct nor reliable indicators of technological efficiency and can be influenced by many factors.

### Enucleation and hemostasis time

**Fig. 1:**
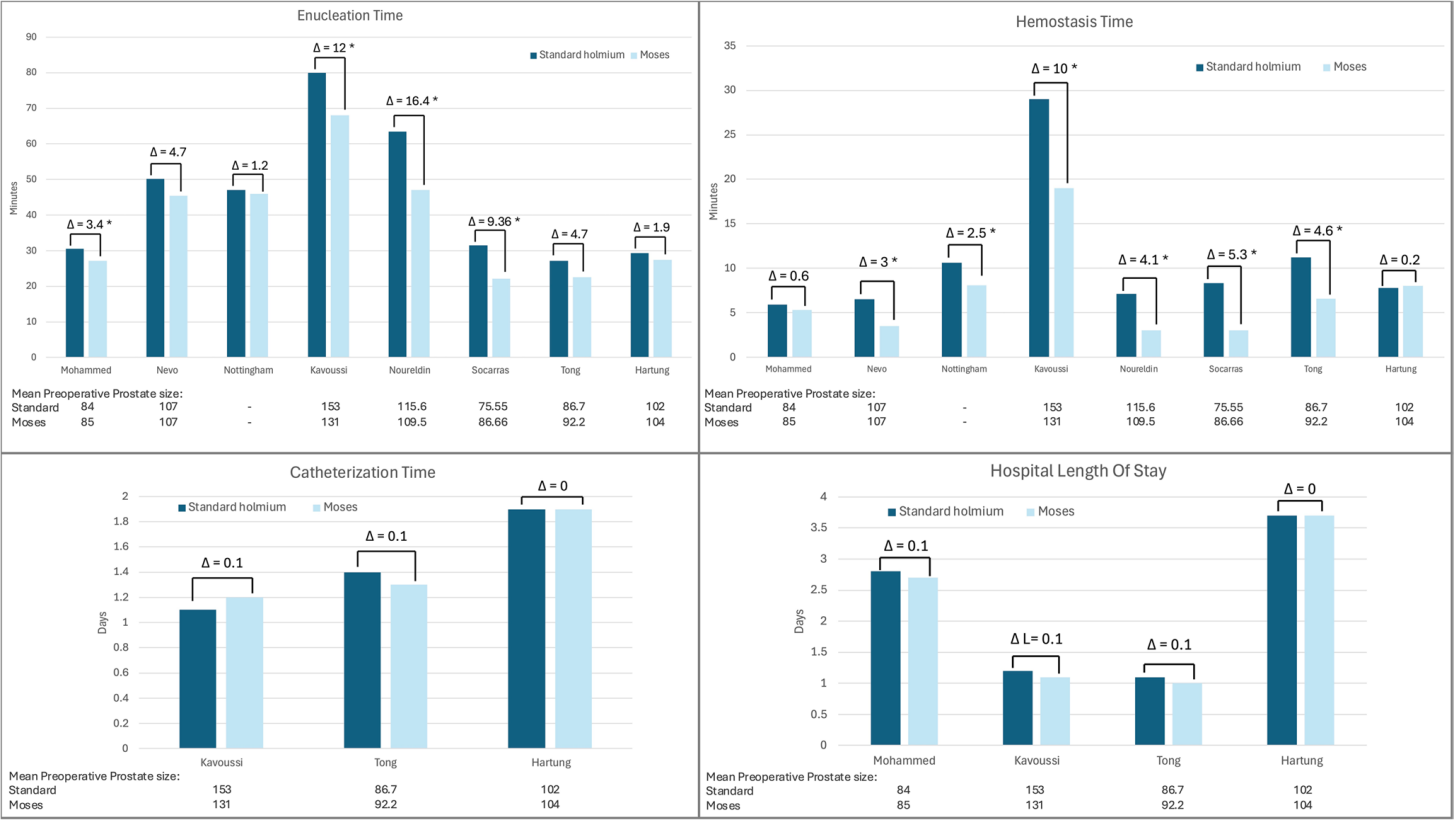
Graphical summary of operative efficiency metrics from studies included in Table 2, showing enucleation time, hospital length of stay, catheterization time, and hemostasis time. * statistically significant. △: difference between values. Four-panel figure comparing operative efficiency outcomes between standard holmium laser (dark bars) and Moses technology (light bars) across multiple studies. The top left panel shows enucleation time (minutes), where Moses is consistently associated with shorter times, with the largest reductions observed in Kavoussi and Noureldin. The top right panel shows hemostasis time (minutes), again demonstrating shorter times with Moses across most studies, particularly in Kavoussi. The bottom left panel shows catheterization time (days), which is similar between groups with minimal differences across studies. The bottom right panel shows hospital length of stay (days), which is also comparable between groups with negligible differences. Across all panels, differences (△) between techniques are displayed above each pair of bars

Although we lacked data on enucleation and hemostasis times, laser energy properties would likely influence both. In this context, while some studies [7, 8, 12, 16] did not notice statistically significant reductions in enucleation time with Moses technology, others [9–11, 15, 17, 18] did find shorter times (Fig. 1). Reported differences in enucleation time range from ~ 2–15 min, with a mean of 39.8 min for standard HoLEP (range 16.4–80) and 38 min for Moses (range 18.1–68) [7–12, 15–18]. This heterogeneity in reported enucleation times suggests that factors beyond pulse modulation may play a role. Additionally, the contribution of hemostasis to operative time may vary depending on surgical technique, as some surgeons perform hemostasis progressively during enucleation while others address bleeding at the end of the procedure, which may influence how operative or enucleation time is recorded.

Holmium laser enables simultaneous tissue cutting and hemostasis. When the laser fiber contacts tissue directly, high-energy density vaporizes the tissue, while peripheral heat dissipates to coagulate adjacent small blood vessels. The effect depends on fiber–tissue distance: direct contact produces cutting and vaporization, whereas a <3 mm distance raises tissue temperature to 60–100 °C, causing protein denaturation and thermal coagulation [19, 20]. Moses pulse modulation is postulated to improve hemostasis by optimizing energy delivery to tissues. To validate this concept, studies began reporting hemostasis time as an efficiency metric. While most series showed a statistically significant reduction with Moses [7–12, 15, 17], some did not [16, 18]. Upon literature review, mean hemostasis was 10.5 min with HoLEP (range 5.9–29) vs. 7 min with Moses (range 3–19) [7–12, 15–18]. Evidently, these differences have been modest and unlikely to translate into meaningful clinical advantages in academic settings with significant trainee involvement.

### Enucleation efficiency

Enucleation efficiency (grams/minute) is a commonly reported performance measure. Although not measured in our cohort, most reports demonstrate higher efficiency with Moses, ranging from 1.1 to 4.11 g/min, compared with 0.7 to 2.6 g/min for holmium [8, 9, 11, 12, 16]. The highest reported value (4.11 g/min) was achieved by an expert with over 9,000 cases, underscoring the influence of surgeon expertise [9]. Although not reported by Kavoussi et al., the calculated mean enucleation efficiency was similar at 0.95 g/min in both arms [15]. Nottingham et al., in contrast, reported higher efficiency with standard holmium (0.73 vs. 0.57 g/min), further highlighting variability across studies [7]. The varying data suggest other factors—surgeon experience, case complexity, and trainee involvement—may play as great a role in efficiency as laser platform selection.

### Postoperative hemoglobin drop and blood transfusion requirements

Our cohort demonstrated comparable outcomes in objective measures of hemostasis, including postoperative hemoglobin drop and transfusion rates. Median hemoglobin drop was 2 g/dL in Moses 1.0 and standard holmium, and 1.7 g/dL in Moses 2.0 (*p* = 0.13), with similar and low transfusion rates (1.6% Moses 1, 0% Moses 2, and 0.5% standard holmium). These findings reinforce that objective bleeding outcomes remain comparable between laser platforms despite theoretical improvements in laser-tissue interaction with Moses technology.

This aligns with broader HoLEP data, where transfusion rates are low, ranging from 0 to 4% [17]. Furthermore, authors comparing standard holmium to Moses have found no differences in transfusion rates, some even reporting 0% [8, 9, 11, 12, 15, 16]. In contrast, hemoglobin drop has shown variability across studies, with some reporting significant differences favoring Moses. Large et al. observed a smaller drop in Moses 1.0 (1 + 1.1 mg/dL) compared with Slimline 550 μm (1.5 + 1.2 mg/dL) and 1000 μm (1.5 + 1.6 mg/dL) holmium groups (*p* = 0.02) [17]. Noureldin et al. similarly found a lower decline with Moses 1.0 (10.7 ± 4.5 g/L vs. 14.7 ± 5 g/L; *p* < 0.001), while Kavoussi reported a smaller hematocrit reduction with Moses 1.0 (-6.4 ± 4.1 vs. -9 ± 4.6; *p* = 0.03) [11, 15]. Others such as Hartung, Socarrás, and Mohamed, noted similar trends though without significance [9, 16, 18]. Even when statistically significant, these differences have been modest and likely not clinically meaningful, reinforcing overall HoLEP safety and suggesting that Moses may offer only incremental improvements in bleeding control. The basis of our findings can be explained by lack in the depth of coagulation necrosis of holmium laser and its Moses pulse modulation in ex-vivo human prostate tissue [21]. Thus, while Moses may improve the intraoperative visual and practical experience of hemostasis (and therefore outpatient feasibility), it does not consistently produce large differences in objective bleeding endpoints.

### Postoperative catheterization time

We observed no significant differences in postoperative catheterization time between groups, consistent with prior studies [7, 9, 15, 16]. Some authors reported improved subjective hemostasis in the postoperative period, enabling same-day catheter removal and same-day discharge (SDD) [10, 22]. However, catheter duration varies widely across studies, ranging from 4 h to 1.9 days, even with Moses technology, reflecting the role of institutional practices [10, 16]. 

### Postoperative hospital stay

Literature generally shows shorter hospital stays with Moses, supported by Gauhar et al.’s meta-analysis [23]. In contrast, we observed shorter stays with standard holmium (1.4 ± 2.1) compared to Moses 1.0 (1.6 ± 0.8) and Moses 2.0 (1.6 ± 0.4), a statistically significant but clinically minor difference. Prior studies show mixed findings, with some reporting shorter stays with holmium and others with Moses, most without statistical significance. Kavoussi et al. similarly reported shorter stays with holmium (1.1 ± 0.5 vs. 1.2 ± 0.63 days), whereas Tong et al. found slightly earlier discharge with Moses (1.0 ± 0.1 vs. 1.1 ± 0.1 days), neither reaching significance [12, 15, 16, 18]. SDD protocols have also been reported with Moses-assisted HoLEP, although these rely on subjective absence of postoperative hematuria during recovery [10, 22]. Overall, variability in hospital stay likely reflects institutional practice patterns and patient preferences, which may explain why the potential benefit of MOSES 2.0 in facilitating same-day discharge is less apparent in settings where overnight observation remains routine.

### Postoperative voiding outcomes

Functional outcomes improved markedly from baseline in all groups, with no significant differences. IPSS improved, Qmax increased, and PVR decreased similarly across platforms. These findings align with prior literature showing comparable symptom and uroflowmetry outcomes between Moses and standard holmium HoLEP [7, 10–12, 15]. Meta-analytic data also support no significant differences in IPSS or Qmax between platforms, although slightly lower PVR has been reported with Moses in some series [24]. Collectively, the evidence supports comparable long-term functional outcomes across all laser platforms.

### Postoperative complications

Although not statistically significant, Moses 1 showed slightly lower AUR rates on POD1. Reported AUR rates vary widely, ranging from 0 to 5.1% for Moses and 6-10.9% for standard holmium in the literature [9, 12, 16]. Routine PVR assessment after voiding trial at our institution, with re-catheterization offered for PVR > 150 mL, may explain the higher observed rate.

Urinary incontinence is a known complication of endoscopic prostate enucleation, which can have substantial impact on patient satisfaction [25, 26]. Prior studies report similar 1-month incontinence rates across platforms (~ 5–7% SUI), although leakage severity and pad use were not consistently reported [10, 12]. Our higher rates likely reflect our strict definition of incontinence as any postoperative leakage requiring protection.

### Limitations

This study has several limitations. First, its retrospective design limits causal inference. Second, the absence of granular intraoperative timing variables, including enucleation and hemostasis time, precludes mechanistic evaluation of laser-dependent procedural phases. Third, OR time may be influenced by non-surgical factors such as anesthesia practices, room turnover efficiency, and institutional workflow patterns. Finally, surgeon experience and case complexity may confound observed differences in OR time. Despite these limitations, OR time remains a clinically meaningful endpoint, and the findings are consistent with prior literature suggesting that improvements in laser efficiency may translate into broader operative workflow benefits.

## Conclusions

HoLEP remains a highly effective surgical option for BPH, with comparable safety and functional outcomes across standard holmium, Moses 1.0, and Moses 2.0 platforms. Although Moses 2.0 may support same-day discharge pathways, its incremental clinical benefit appears limited in healthcare settings where same-day discharge is neither feasible nor routinely pursued. Accordingly, the absence of Moses 2.0 should not be viewed as a barrier to offering HoLEP, as conventional holmium platforms continue to provide excellent and durable outcomes without materially disadvantaging patients. Tailored laser selection based on patient and procedural factors remains essential, and further research is needed to refine optimal laser choice and assess long-term cost-effectiveness.

## Data Availability

All data supporting the findings of this study are available upon request.

## Code availability

All data supporting the findings of this study are available upon request.
